# Synthesis and Performances of Shrinkage-Reducing Polycarboxylate Superplasticizer in Cement-Based Materials

**DOI:** 10.3390/ma15197002

**Published:** 2022-10-09

**Authors:** Shiyu Li, Xiao Liu, Yurui Xu, Guanghong Lai, Yungchin Ding, Yichen Chen, Chunlei Xia, Ziming Wang, Suping Cui

**Affiliations:** 1 Key Laboratory of Advanced Functional Materials, Ministry of Education, Faculty of Materials and Manufacturing, Beijing University of Technology, Beijing 100124, China; 2 Institute of Mineral Resources Engineering, National Taipei University of Technology, Taipei 10608, China; 3 Materials Technology Center, Beijing Municipal Engineering Research Institute, Beijing 100037, China; 4 National Engineering Laboratory for Industrial Big-Data Application Technology, Faculty of Materials and Manufacturing, Beijing University of Technology, Beijing 100124, China

**Keywords:** shrinkage-reduction, polycarboxylate superplasticizer, molecular design, mortar, synthesis

## Abstract

Reducing or eliminating cracks caused by shrinkage of cementitious materials remains a daunting challenge for construction engineers. Drying shrinkage and autogenous shrinkage are the main shrinkage types in the service process of cement-based materials, which have a great impact on engineering applications. If cracks in concrete generate by drying or autogenous shrinkage, the mechanical properties, water resistance and durability of concrete will be also affected. It is an effective method to use chemical admixtures to inhibit the shrinkage of cement-based materials. Polycarboxylate plasticizer (PCE) is an important chemical admixture in cement-based materials and is widely used in practical engineering. It can bring great value by reducing the shrinkage effect through molecular design. Through our innovative design, a series of shrinkage-reducing polycarboxylate superplasticizers (SRPs) were synthesized, their molecular structures were confirmed by Fourier transform infrared spectroscopy (FTIR) and their molecular properties were determined by gel permeation chromatography (GPC). Furthermore, the shrinkage performances at different ages of the mortars containing the synthesized SRPs with different structures were systematically evaluated. The results showed that compared with the blank sample, the dry shrinkage rate and free shrinkage rate of the mortars containing SRP decreased by over 20% and 15%, respectively. Additionally, the shrinkage rates of the mortars containing SRP were significantly lower than that of the mortar containing conventional PCE, and moreover, the water-reducing performance was improved compared to conventional PCE. Based on the experimental results of surface tension and evaporation rate of different SRP solutions, the mechanism of the shrinkage-reducing effect was probed, as expected to provide guidance for the design and development of new shrinkage-reducing admixtures.

## 1. Introduction

The shrinkage of cement-based materials has been the focus of engineers and academics. Shrinkage is an inherent characteristic of cement-based materials, taking place both in the plastic and hardened stages. When concrete is used in building structures, it will be restricted by many factors, such as steel or adjacent structures. Under both the influence of the shrinkage process and external constraints, the concrete matrix usually develops harmful cracking [1,2,3]. Due to shrinkage cracking, a pathway is created for the penetration of aggressive agents (i.e., chlorides, sulfate) into the cement-based materials, thereby deteriorating the long-term durability and serviceability of the concrete structure [4,5,6]. Shrinkage cracking is considered the most challenging problem in all stages of the entire service life of concrete and may cause serious damage to concrete structures. The resulting costs related to maintenance are relatively high and will cause more energy consumption and carbon emissions [7,8]. The shrinkage of cement-based materials is generally divided into plastic shrinkage, autogenous shrinkage, drying shrinkage, temperature shrinkage and carbonization shrinkage. Drying shrinkage and autogenous shrinkage are the main shrinkage types of cement-based materials. The basic mechanism of shrinkage is that the volume of the microstructure in the cement matrix decreases due to water loss [2,3]. These two kinds of shrinkage occur continuously in the service process of cement-based materials and have a great impact on engineering applications.

Chemical admixture, as an indispensable component of modern concrete, is widely used in construction engineering. Superplasticizer is a widely used and studied type of chemical admixture and it is usually used to reduce water consumption and adjust the workability of concrete [8,9,10]. Thus, it plays a key role in the development and application of modern high-performance concrete. However, the inhibition of the current conventional superplasticizer on the shrinkage and cracking of concrete is weak, and at low water–binder ratio, the shrinkage and cracking are more likely to be caused under dry conditions [11,12,13,14]. Under the same fluidity or slump, superplasticizers with different molecular types and structures had different effects on the resistance of shrinkage and cracks in concrete. In the family of superplasticizers, naphthalene superplasticizers, sulfamate superplasticizers and aliphatic superplasticizers can significantly increase the early shrinkage and total shrinkage of concrete [14,15]. Relatively, polycarboxylate superplasticizer (PCE), the latest generation of superplasticizer, performs better than other types of superplasticizers, but its impact on concrete shrinkage is still complex in general. Different reports may have opposite results and the possibility of PCE reducing or increasing the early shrinkage and total shrinkage of concrete exists [11,12,13,14,16]. Since superplasticizer is very important for the preparation of concrete, the situation that superplasticizer increases the shrinkage of concrete has attracted widespread concerns among researchers and engineers.

In the preparation process of concrete, if shrinking-reducing agents (SRAs) are added to the slurry according to the actual requirements to inhibit the shrinkage of concrete, it will increase the complexity of the preparation process and the problem of compatibility between other admixtures and SRAs, which may be detrimental to the mechanical properties of the concrete [17,18,19]. In this case, it is urgent to develop a new type of superplasticizer with a shrinkage-reduction effect. PCE is favored by researchers because of its excellent performance and strong molecular designability. Researchers hope to use some monomers with shrinkage-reducing ability to modify PCE molecules and prepare a new type of PCE with shrinkage-reducing efficiency, namely shrinkage-reducing polycarboxylate superplasticizer (SRP) [8]. As a functional PCE, SRP not only has the advantages of high water-reduction and strong dispersion performance but also has the superiority of reducing shrinkage of cement-based materials. In recent years, SRP has attracted significant attention and some patents [20,21,22,23,24] and research results have been publicly reported [25,26]. In the current research, the shrinkage-reducing monomers used in the preparation process of SRP were usually obtained by the esterification of unsaturated carboxylic acid and alcohol-based shrinkage-reducing components. This preparation process is natural and reasonable. In SRAs used to inhibit drying shrinkage and autogenous shrinkage, alcohols are commonly used substances. Their mechanism is to reduce the capillary pressure of pores of cement matrix during water loss by reducing the surface tension of cement pore solution, thereby reducing shrinkage. Therefore, it is necessary to select an alcohol with a specific structure and low surface tension when designing the reducing monomer and esterify the alcohol with unsaturated acid to prepare the reducing monomer with polymerization activity. The unsaturated ester formed by esterification was polymerized with other monomers of PCE through free radical polymerization to achieve the purpose of introducing shrinkage-reducing components into the molecular structure. When the types and dosages of unsaturated carboxylic acid and alcohol-based compounds change, this corresponds to the changes in the molecular structure of SRP and further shrinkage-reducing performances. However, there are few studies on the influence of SRP molecular structure on shrinkage-reducing performances and mechanisms.

In this study, a series of SRPs with different molecular structures were successfully designed and synthesized by using new monomers and then their molecular structures and molecular weights were characterized. Additionally, these synthesized SRPs with different molecular structures were applied to test the fluidity properties of cement pastes and the shrinkage properties of mortars at different ages. The mechanism of the shrinkage reduction was probed by the evaluations of surface tension and evaporation rate of solution with SRPs. The aim of this study is to clarify the influence of molecular structures of SRP on shrinkage performances of cement-based materials, providing a direction for designing the molecular structure of SRP to further improve shrinkage-reducing effectiveness.

## 2. Materials and Methods

### 2.1. Materials

Acrylic acid (AA), maleic anhydride (MA), mercaptoacetic acid (TGA), ammonium persulfate (APS), p-toluenesulfonic acid (p-TsOH) and toluene (all ≥ 98% purity) were obtained from Tianjin Guangfu Fine Chemical Research Institute (Tianjin, China). Isopentenol polyoxyethylene ether macromonomer (TPEG) was provided by Liaoning Oxiranchem, Inc (Liaoning, China). Reference cement P·I·42.5 was supplied by China Building Materials Research Institute (Beijing, China), and its chemical and mineral compositions are given in Table 1. ISO standard sand was produced by Xiamen ISO Standard Sand Co., Ltd. (Xiamen, China). The sand with a fineness modulus of 2.7 has a density of 2650 kg/m^3^ and a bulk density of 1460 kg/m^3^.

### 2.2. Synthesis

#### 2.2.1. Synthesis of Shrinkage-Reducing Monomer

Shrinkage-reducing monomers were synthesized by the esterification of carboxylic acid and functional alcohols under the condition of catalyst and heating. The difference between functional alcohols is that the structural content of EO and PO in the molecule is different. The synthesized shrinkage-reducing monomers were identified as SRM-1, SRM-2 and SRM-3. Both SRM-1 and SRM-3 contained the structure of acrylic acid but the functional alcohol structure was different, while SRM-2 contained the structure of maleic acid and the functional alcohol structure, which was the same as that of SRM-3.

During the synthesis of shrinkage-reducing monomers, unsaturated carboxylic acids and alcohol-based compounds were added to the flask equipped with the condenser. In this study, p-TsOH was used as catalyst in the esterification reaction to reduce the by-products. Since the esterification reaction is reversible, it was also necessary to add toluene, a low boiling solvent, and form an azeotrope to remove the water, so that the esterification reaction was carried out in a positive direction. In order to ensure the esterification rate was as high as possible, the reaction temperature was controlled at 100–130 °C, and the reaction time was about 4–6 h. During the reaction, some solution was taken from the flask and the esterification rate of the reactants in the system was determined by titration. When the esterification rate was stable to equilibrium, the reaction was terminated. The calculation of the esterification rate is as shown in Formula (1). After the reaction, the product was cooled and transferred to the storage container:

(1) $$\mathrm{Esterification}\;\mathrm{rate}=\frac{H_{0}-H_{t}}{H_{\mathrm{theo}}}\times 100\%$$

where H_0_ is the initial acid value; H_t_ is the acid value of the system at t min; and H_theo_ is the theoretical acid value in which an alcohol-based compound is completely esterified.

#### 2.2.2. Synthesis of SRP

The shrinkage-reducing monomer obtained from Section 2.2.1 and TPEG macromonomer were mixed with distilled water in a four-neck round-bottom flask which was placed in a constant temperature bath at 65 °C with stirring until monomer completely dissolved. Thereafter, AA, APS and TGA aqueous solutions were added dropwise to the flask within 2 h, respectively. After the dropping of aqueous solutions was completed, the reaction lasted at a constant temperature of 65 °C with stirring for 1h, and then was cooled to room temperature to obtain the final product. The detailed composition of the synthesized SRPs is shown in Table 2.

### 2.3. FTIR Analysis

The samples of SRP were placed in a drying oven (at 105 °C for over 2 h) to remove the free water to prevent hydroxyl groups in water molecules from affecting the sample spectrum, and the solid SRPs were then mixed with potassium bromide and pressed as mixed powder into a transparent disk by tablet press. FTIR spectra of samples were conducted on VERTEX 70 Fourier transform infrared spectrometer (Bruker Co., Rheinstette, Germany). The measurement range of wavenumber was from 500 to 4000 cm^−1^.

### 2.4. GPC Analysis

The number average molecular weight (M_n_), weight average molecular weight (M_w_) and polydispersity index (PDI) of specimens were determined on a Waters PL-GPC50 (Polymer Laboratories, London, UK). Completely dried solid samples of PCE or SRP were dissolved in sodium nitrate aqueous solution whose concentration was 0.1 mol/L, and the concentration of polymer in the solution was controlled within the range of 1–3 g/L. GPC equipped with a PL aquaqel-OH MIXED 8 μm chromatographic column, laser light scattering (LS) and refractive index (RI) detectors, was used to characterize the molecular weight of polymers. The eluent was also aqueous solution of sodium nitrate with an injection volume of 100 μL and a flow rate of 1.0 mL/min, and the elution time of the sample was 30 min.

### 2.5. Paste Fluidity Test

The experiments on the fluidity of cement pastes containing polymer were carried out according to the Chinese standard GB/T 8077-2012. The dosage of admixture was 0.2% by weight of cement and the water–cement ratio (W/C) was 0.29, and the water in the admixture solution should be deducted when the mixing water was weighed. The detailed mix proportion of cement pastes is shown in Table 3. The cement paste was mixed at a low speed (140 r/min) for 2 min and then at a high speed (280 r/min) for another 2 min. Then, paste was transferred into a mini-slump cone (60 mm height, 36 mm top diameter and 60 mm bottom diameter), which was then slowly removed vertically. After 30 s, the length of two maximum spread diameters perpendicular to each other was measured, and the average value was recorded as the initial fluidity. To measure the fluidity retention, the pastes were transferred to the mixing vessel again and sealed with a wet towel to retain the moisture state. After 1 and 2 h, the mixture was stirred at high speed (280 r/min) for 1 min and then the fluidity value was determined again by repeating the process above.

### 2.6. Shrinkage Test 

Shrinkage tests of mortars containing PCE or SRP were conducted according to Chinese standard JC/T 603-2004.

The ratio of cement to standard sand in the mortar was 1:2 (mass ratio) and the water consumption was determined by the fluidity of the mortar reaching 130–140 mm. The fluidities of mortars were tested according to Chinese standard GB/T 2419-2016, with the admixture dosage of 0.15% (by weight of cement).

The specimens used in the shrinkage tests were demolded (the mold size was 25 mm × 25 mm × 280 mm) after being cured for 24 ± 2 h at a temperature of 20 ± 1 °C and relative humidity of more than 90%. The specimens were cured or dried as required after demolding, and the specimens for dry shrinkage should be cured for 7 d, while the specimens for free shrinkage should be dried directly. Three samples are tested for each composition.

In the drying shrinkage test, the initial lengths of specimens were measured by comparator and transferred specimens to the drying chamber with a temperature of 20 °C ± 3 °C and relative humidity of 50% ± 4%. Then, the lengths for 7 d, 14 d, 21 d and 28 d were measured and the relative shrinkage reduction rates of mortars with PCE or SRP were calculated according to Formula (2):

(2) $$\mathrm{RDS}=\frac{L_{s0}-L_{\mathrm{sn}}}{L_{B0}-L_{\mathrm{Bn}}}\times 100\%$$

where RDS is the relatively dry-shrinkage rate; L_s0_ and L_sn_ are the initial length and the n day length of the mortar with PCE or SRP, respectively (mm); L_B0_ and L_Bn_ are the initial length and the n day length of the blank mortar (mm).

In the free shrinkage test, the initial lengths of specimens were measured by comparator and transferred specimens to the drying chamber immediately after being demolded. Then, the lengths for 2 d, 3 d, 7 d, 14 d, 21 d and 28 d were measured and the relative shrinkage reduction rates of mortars with PCE or SRP were calculated according to Formula (3):

(3) $$\mathrm{RFS}=\frac{l_{s0}-l_{\mathrm{sn}}}{l_{B0}-l_{\mathrm{Bn}}}\times 100\%$$

where RFS is the relatively free-shrinkage rate; l_s0_ and l_sn_ are the initial length and the n day length of the mortar with PCE or SRP; respectively (mm); and l_B0_ and l_Bn_ are the initial length and the n day length of the blank mortar (mm).

### 2.7. Surface Tension Test

The surface tension values of polymer aqueous solution were measured by a BZY-1 Surface or interface tension apparatus produced by Shanghai Hengping Instrument Factory (Shanghai, China). The concentration of aqueous solution of PCE or SRP was 10 wt%. The platinum plate method was used for the test. When the platinum plate was immersed in the sample solution, it was affected by the surface tension. Until the surface tension of the solution was balanced with the external force provided by the instrument, the platinum plate stopped immersion into the liquid. Then, the balance sensor of the instrument measured the immersion depth and converted it into the surface tension value of the solution.

### 2.8. Evaporation Rate Test

The evaporation rate of polymer solution was calculated based on the mass loss of solution. The aqueous solution of PCE or SRP with a concentration of 10 wt% was placed in an evaporating dish and evaporated in a constant-temperature oven with a constant wind speed of 30 °C. The mass was recorded at 24 h and 48 h, respectively, and the evaporation rate was calculated according to Formula (4):

(4) $$\mu =\frac{m_{0}-m_{t}}{S\cdot t}$$

where μ is the evaporation rate (×10^−3^ g/cm^2^·h); m_0_ and m_t_ are the initial mass of the solution and the mass of the solution for t h, respectively (g); S is the area of the evaporating dish (cm^2^); and t is the test time of the solution (h).

## 3. Results and Discussion 

### 3.1. Characterization of SRM and SRP

The final esterification rates of the three types of shrinkage-reducing monomers are given in Table 4. These esterification rates were obtained under the conditions of a reaction temperature of 130 °C and a reaction time of 5 h. It can be seen that there are high esterification rates of the three shrinkage-reducing monomers, indicating that the reaction degree was higher and the amount of by-product was less. This proves that the preparation process was highly effective and the high yield was also helpful for the synthesis of SRP.

**Figure 1:**
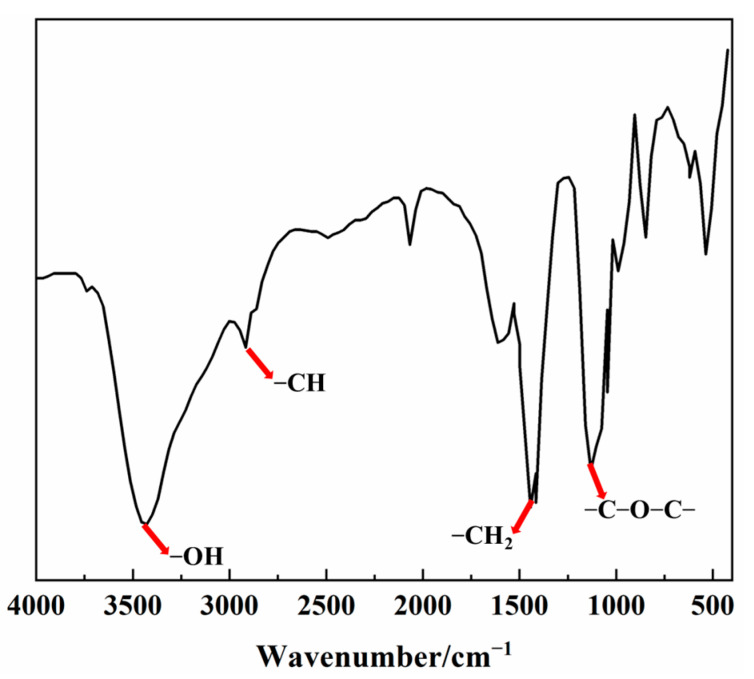
FTIR spectrum of the synthesized SRP-1.

The FTIR spectrum of the SRP is shown in Figure 1. Due to the similarity of the structures and functional groups in the three SRPs molecules, SRP-1 was selected as the representative specimen.

From the FTIR spectrum, a wide and strong peak appears at around 3430 cm^−1^, indicating that the sample contains a large number of intramolecular hydrogen bonds, which are formed by the hydroxyls at the end of TPEG side chains. There are two stretching peaks at around 2920 cm^−1^ and 1465 cm^−1^, which confirms the existence of -CH and -CH_2_, respectively. Distinguishingly, a peak appears at around 1131cm^−1^ corresponding to the ester group, which is the characteristic functional group of SRPs [27,28,29]. All of the results thus show that the SRPs have various functional groups including common groups of PCE as well as exclusive ester groups, which are consistent with the designed molecular structure, proving the successful preparation of SRP.

Table 5 shows the molecular weight results of three types of SRP by GPC analysis. It can be seen from Table 5 that the molecular weights of three SRPs are all higher than that of PCE because the molecular weights of the shrinkage-reducing monomers are higher than that of AA, especially for the highest molecular weight of the shrinkage-reducing monomer used in SRP-2, which makes its molecular weight significantly higher than the others. The significant increase in molecular weight effectively proves the polymerization of the shrinkage-reducing monomer with AA and TPEG. In addition, the introduction of a shrinkage-reducing monomer in SRP seems to improve the degree of polymerization. According to the theoretical calculation of molecular structures, the M_w_ of SRP1-3 should be about 4%, 11% and 5% higher than that of PCE, respectively, and thus, from the M_w_ results of SRP1-3 in Table 4, the M_w_ of PCE should be in the range of 53,000–55,500 g/mol. However, it can be seen in Table 4 that the measured values of molecular weight of PCE were lower, indicating that the polymerization degree of monomers of PCE may be lower than that of SRP, which can also be indicated by the value of PDI. The PDI values of SRP were significantly lower than that of PCE, indicating that the molecular weight distribution was narrower and there were fewer free monomers or low molecular weight polymers in the system. The results of GPC and FTIR analysis further indicate that the target of synthesis was achieved.

### 3.2. Fluidity

**Figure 2:**
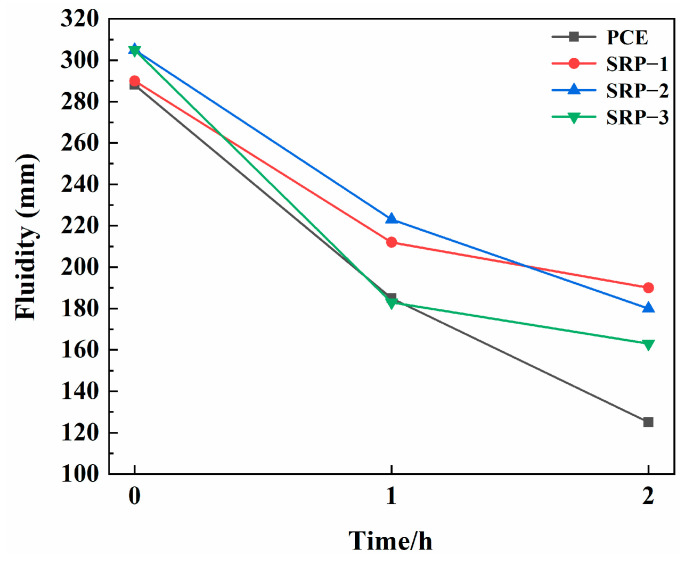
Fluidities and fluidity retentions of cement pastes containing PCE or SRP.

Figure 2 shows the fluidities and fluidity retentions after 1 h and 2 h of cement pastes containing PCE or SRP. From Figure 2, the fluidities of cement pastes with SRP were entirely higher than that of cement pastes with PCE, and the ability of SRP to retain the fluidity of cement paste within 2 h was also 37.2% higher than that of PCE. These results show that compared with PCE, SRP can exhibit a lower dosage when reaching the equivalent paste fluidity, and the fluidity retention of cement pastes containing SRP was better, showing higher working effectiveness. Consequently, partially replacing AA with the shrinkage-reducing monomer cannot weaken the dispersing capacity, even slightly enhanced. For these shrinkage-reducing monomers, SRM-2 performed better, for which the corresponding SRP shows better initial fluidity and fluidity retention of cement paste.

The improvement of the fluidity retention ability of cement paste with SRP can be expected and easily understood. In the environment of the high pH of a cement solution, some ester groups of SRPs were hydrolyzed gradually, which produced more anionic charges in the molecules, and these new anionic charges continued to adsorb on the surface of cement particles to retain the dispersion of cement paste. This had been confirmed many times in related studies [30,31,32,33]. The hydrolysis process of SRP-2 molecules could expose more negatively charged carboxyl groups so that its fluidity retention ability was the best. This directly confirms the rationality of this assumption. Interestingly, the effect of SRP on the improvement of initial fluidity may be more novel, and the research of ManuelIlg et al. [34] can provide some help for the discussion of this phenomenon. ManuelIlg et al. reported that non-adsorbing small molecule alcohol-based compounds with low molecular weight could be used as auxiliary dispersants to enhance the dispersion effect of PCE. The ester groups in SRP could be slightly hydrolyzed at the initial stage of cement mixing and a small amount of alcohol-based components would be released to cooperate with SRP molecules to improve the initial fluidity of cement paste.

### 3.3. Drying Shrinkage

**Figure 3:**
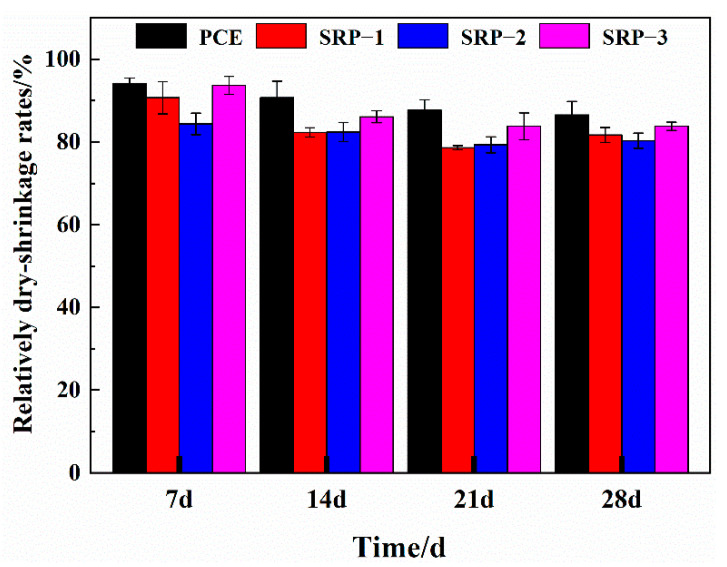
Dry-shrinkage rates of mortars containing PCE or SRP.

The drying shrinkage rates of mortars containing PCE or SRP at different ages are shown in Figure 3. From the experimental results, the drying shrinkages of the mortars containing PCE or SRP at all ages were lower than that of the blank sample. Additionally, in Figure 3, it is apparent that the mortars containing SRP at all ages exhibited a lower relatively dry-shrinkage rate than that containing PCE, which is caused by the role of SRP employing a shrinkage-reducing monomer. In comparison, the mortar containing SRP employing an SRM-22 monomer exhibited a relatively dry-shrinkage rate of about 20%, showing a better shrinkage-reducing ability. This indicates that the shrinkage-reducing monomer containing a diester structure can produce a better dry-shrinkage reduction effect. However, comparing the reduction effects of SRP-1 and SRP-3 on the dry-shrinkage of mortar, it can be seen that under the same unsaturated carboxylic acid monomer, the change of the alcohol-based shrinkage-reducing monomer has a certain influence on the shrinkage-reducing effect of SRP.

### 3.4. Free Shrinkage

**Figure 4:**
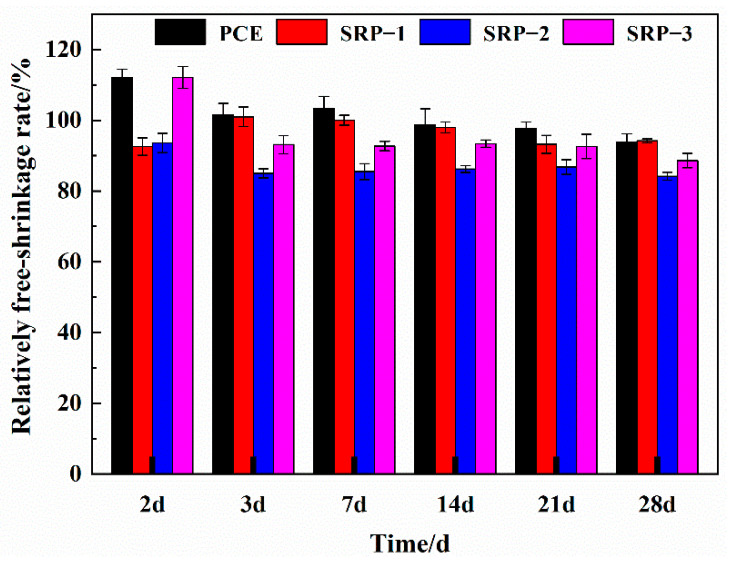
Free-shrinkage rates of mortars containing PCE or SRP.

The free shrinkage rates of mortars containing PCE or SRP at different ages are shown in Figure 4. It can be seen from Figure 4 that, at an early stage (2 d, 3 d and 7 d), the relatively free shrinkage rates of mortars with PCE, SRP-1 or SRP-3 were higher than 100%, while after 14 days, the relatively free shrinkage rates of mortars containing PCE or SRP decreased to less than 100%. The free shrinkage of mortar includes autogenous shrinkage and drying shrinkage [1,3]. It was confirmed in Section 3.3 that the shrinkage-reducing monomer has a significant reduction effect on the drying shrinkage of mortar but the results of this section show that the shrinkage-reducing monomer may not have such a significant reduction effect on the autogenous shrinkage of mortar at an early age. In general, the free shrinkage rates of mortars with SRP at different ages were lower than that of mortars with PCE, indicating that SRP has obvious advantages over conventional PCE. It is worth noting that SRP-2 containing a diester structure still has the most significant effect on reducing the free shrinkage of mortar, showing the positive potential in being applied as a shrinkage-reducing material. 

### 3.5. Surface Tension

**Figure 5:**
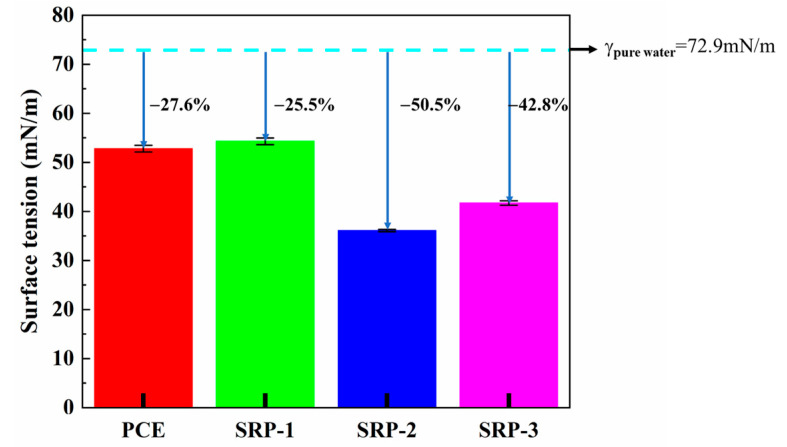
Surface tensions of PCE and SRP solutions.

**Figure 6:**
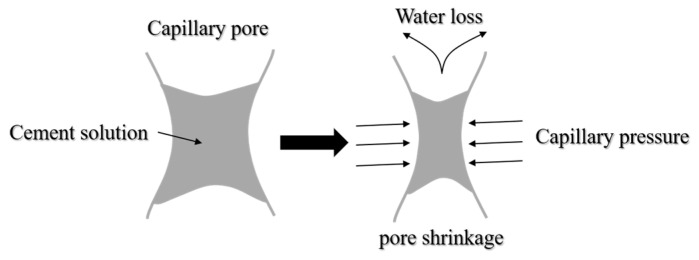
Schematic diagram of drying shrinkage of capillary of cement-based materials.

The surface tension values of the PCE and SRP solution are shown in Figure 5. It can be seen from Figure 5 that the surface tension values of the PCE and SRP were in the range of 35–55 mN/m, which were lower than that of pure water (γ_pure water_ = 72.9 mN/m). This shows that PCE and SRP can significantly reduce the surface tension of the aqueous solution, which is beneficial to the wetting, osmotic adsorption of the surface of cement particles and foaming on the gas–liquid interface [35,36]. The reduction of surface tension in cement solution is very critical in reducing the drying shrinkage of mortar. When the relative humidity of the external environment is lower than 100%, the free water inside the capillary pores of mortar evaporates and escapes into the environment, and the surface of the cement solution drops to form a meniscus. The capillary pressure generated in these pores deforms the mortar and causes drying shrinkage [35,36], as shown in Figure 6. The relationship between the capillary pressure and the surface tension of the pore solution, under a certain pore diameter, can be described by the Young–Laplace equation, as shown in Formula (8) [17,37,38].

(5) $${}_{\mathrm{cap}}=\frac{2\gamma \cos\theta }{R}$$

where σ_cap_ is the capillary pressure; γ is the surface tension of solution (mN/m); θ is the contact angle between the solution and the cement matrix; and R is the diameter of the capillary pore.

It can be seen that reducing the surface tension of cement solution helps to reduce the capillary pressure, thereby reducing the drying shrinkage of the mortar. Combined with the results mentioned above, SRP-2 showed the lowest surface tension, which is completely consistent with the maximum reduction of drying shrinkage of mortar.

### 3.6. Evaporation Rate

**Figure 7:**
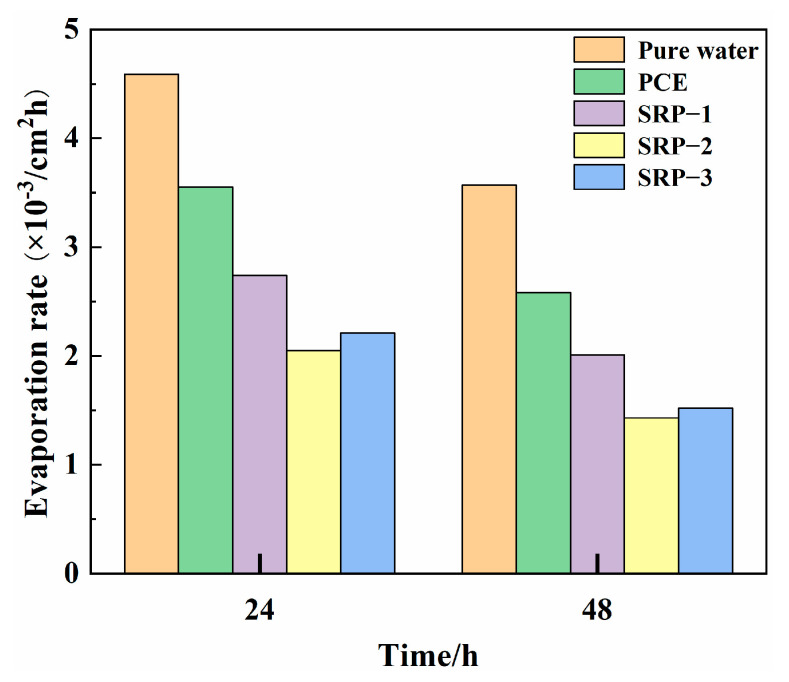
Evaporation rates of PCE and SRP solutions at different times.

The evaporation rates of the PCE and SRP solutions within 24 h and 48 h are shown in Figure 7. Figure 7 shows that the evaporation rate of the solution could be reduced by adding PCE or SRP. The evaporation rates of PCE and SRP solutions within 24 h and 48 h were significantly lower than that of pure water. In addition, SRP solution, especially SRP-2 solution, exhibited the lowest evaporation rate within both 24 h and 48 h, showing that the evaporation rates of 24 h and 48 h were 56.6% and 57.4% lower than that of pure water, respectively, which manifests as the best water-retaining property. This is because the water molecules in the solution form hydrogen bonds with the ether bonds in the SRP molecules and the small molecular alcohols generated by hydrolysis [39,40,41], which limits the movement of water molecules and increases the energy barrier for water molecules evaporating into the environment. In addition, SRP also has a significant effect on the colligative properties of the solution, which can effectively reduce the boiling point of the solution and inhibit the evaporation of water. Therefore, the addition of SRP can make the cement pore solution difficult to evaporate, reducing the capillary pressure and further reducing the shrinkage.

### 3.7. Holistic Analysis

The independent discussion in each section may not be able to comprehensively explain the differences in the mechanism between PCE and SRP. In this section, the differences between PCE and SRP in the mechanism of reducing shrinkage of mortar are systematically described by combining previous discussions with the current public literature. Although in this study PCE showed some shrinkage reduction effects in cement-based materials, it can be seen from Section 3.4, Section 3.5 and Section 3.6 that SRP had more significant effects on reducing the shrinkage of mortar and changing the properties of the solution.

In Section 3.5 and Section 3.6, it was discussed that SRP reduced the surface tension and evaporation rate of the solution, which was the key to reducing mortar shrinkage. However, it should be noted that PCE also reduced the surface tension and evaporation rate of the solution but its shrinkage reduction effect was not significant compared with SRP, and even showed the effect of shrinkage increase in some reports. Therefore, it needs to be further discussed. PCE usually plays a role in cement slurry by adsorbing on the surface of cement particles [42,43,44]. In the actual slurry, not all PCE molecules can be dissociated in the solution, and PCE has a limited effect on reducing the surface tension and evaporation rate of the solution compared with the ideal environment in Section 3.5 and Section 3.6. In addition, with the hydration process of cement, many PCE molecules adsorbed on the surface of cement particles are fettered to the cement matrix [45,46], which further reduces the concentration of PCE in the pore solution. On the other hand, studies have shown that PCE can promote the hydration rate of cement [47], which consumes the free water in the cement matrix, thereby further weakening the potential shrinkage reduction effect of PCE.

As for SRP, as described in Section 3.2, the shrinkage-reducing monomers in the SRP molecules partially hydrolyze in the alkaline environment of cement solution to release non-adsorbing small molecule alcohol-based compounds [30,31,32,33,48]. Although SRP has almost the same backbone structure as PCE and has a similar process to PCE in cement paste, the alcohol-based compounds released from the SRP molecules are less fettered in the cement matrix [34]. On the contrary, these alcohols are free in the pore solution, making the state of the pore solution closer to the ideal state set in the experiment.

The difference in the molecular structure of SRP also profoundly affects its shrinkage reduction effect. Comparatively, SRP-2 with a diester structure performs better. This is logical. Compared with SRP-1 and SRP-3, SRP-2 containing a diester structure in cement paste can hydrolyze to produce alcohol-based compounds with a higher concentration, which can change the properties of the cement pore solution and reduce the shrinkage of cement-based materials. In addition, different types of alcohol-based compounds also have different effects on the shrinkage reduction of SRP. It can be seen that the shrinkage reduction effect of the diester structure is significantly superior to that of the monoester structure, and this substantial result is still worthy of further attention.

## 4. Conclusions

The focus of this study is to synthesize a series of SRPs with different molecular structures and investigate their effects on the shrinkage-reducing performance of cement mortar and its mechanism. The main conclusions are as follows.

(1) The molecular structure and molecular weight of the synthesized SRPs were characterized by FTIR and GPC analysis, which confirmed the achievement of the synthesis target and the rationality of the synthesis process;
(2) Compared with conventional PCE, SRP showed the improved fluidity of cement paste, and the fluidity retention ability was increased by 37.2%, indicating that partially replacing AA with the shrinkage-reducing monomer could enhance the dispersing capacity;
(3) Compared with pure water, the surface tension and evaporation rate (within 48 h) of an SRP aqueous solution can be reduced by 50.5% and 57.4%, respectively. SRP effectively reduced the shrinkage of mortar through the combination of two mechanisms, i.e., reducing the surface tension of cement solution in capillary pores and inhibiting the evaporation of water in the solution. This reduction was very significant, especially for drying shrinkage which decreased by over 20%;
(4) The diester structure in SRP molecules showed greater advantages in improving the fluidity of cement paste and reducing the shrinkage of cement-based materials, which was related to the hydrolysis process of ester groups in the molecules and the non-adsorbing small molecule alcohol-based compounds produced by hydrolysis;
(5) The new type of SRP developed in this study can be used as a functional material in cement-based materials and has a potential application in concrete engineering requiring low shrinkage, high workability and long fluidity retention.

The above research results fully demonstrated the great advantages of SRP and verified the influence of different molecular structures on the shrinkage-reducing effect. These findings are expected to contribute to future research in this field and promote the application of SRP, which also helps to improve the durability and safety of building structures and reduce unnecessary government expenditure and carbon emissions. Future research should concentrate on the adsorption process, hydrolysis kinetics and effects on hydration and pore structure of SRP, which will help to further understand the effects of SRP on various shrinkage processes.

## Figures and Tables

**Table 1:** Chemical and mineral compositions of reference cement.

**Chemical composition (wt %)**							
**SiO_2_**	**Al_2_O_3_**	**Fe_2_O_3_**	**CaO**	**MgO**	**SO_3_**	**Na_2_Oeq**	**f-CaO**
22.93	4.29	2.89	66.23	1.92	0.35	0.70	0.64
**Mineral composition (wt %)**							
**C_3_S**	**C_2_S**	**C_3_A**	**C_4_AF**				
58.78	21.38	6.49	8.77				

**Table 2:** Composition of the synthesized SRPs.

Sample	Shrinkage-Reducing Monomer	Composition (Molar Ratio)		
		AA	Shrinkage-Reducing Monomer	TPEG
PCE	-	3	0	1
SRP-1	SRM-1	2.5	0.5	1
SRP-2	SRM-2	2.5	0.5	1
SRP-3	SRM-3	2.5	0.5	1

**Table 3:** Mix proportions of cement pastes.

Sample	Cement/g	Water/g	PCE/g	SRP/g
PCE	300	87	0.6	-
SRP-1			-	0.6
SRP-2			-	0.6
SRP-3			-	0.6

**Table 4:** Esterification rates of the three types of shrinkage-reducing monomers.

Sample	SRM-1	SRM-2	SRM-3
**Esterification rates/%**	90.95	92.04	91.80

**Table 5:** Molecular properties of the synthesized SRPs.

Sample	PCE	SRP-1	SRP-2	SRP-3
**M_n_**	16,163	24,797	27,158	23,127
**M_w_**	43,201	57,645	59,917	56,154
**M_z_**	94,777	111,245	135,427	104,993
**PDI**	2.67	2.32	2.21	2.43

## Data Availability

The data presented in this study are available on request from the corresponding author.
